# Insights into the Roles of Gut Microbes in Obesity

**DOI:** 10.1155/2008/829101

**Published:** 2008-12-03

**Authors:** Yolanda Sanz, Arlette Santacruz, Giada De Palma

**Affiliations:** Institute of Agrochemistry and Food Technology (IATA), Spanish National Research Council (CSIC), P.O. Box 73, Burjassot, 46100 Valencia, Spain

## Abstract

Obesity is a major public health issue as it enhances the risk of suffering several chronic diseases of increasing prevalence. Obesity results from an imbalance between energy intake and expenditure, associated with a chronic low-grade inflammation. Gut microbes are considered to contribute to body weight regulation and related disorders by influencing metabolic and immune host functions. The gut microbiota as a whole improves the host's ability to extract and store energy from the diet leading to body weight gain, while specific commensal microbes seem to exert beneficial effects on bile salt, lipoprotein, and cholesterol metabolism. The gut microbiota and some probiotics also regulate immune functions, protecting the host form infections and chronic inflammation. In contrast, dysbiosis and endotoxaemia may be inflammatory factors responsible for developing insulin resistance and body weight gain. In the light of the link between the gut microbiota, metabolism, and immunity, the use of dietary strategies to modulate microbiota composition is likely to be effective in controlling metabolic disorders. Although so far only a few preclinical and clinical trials have demonstrated the effects of specific gut microbes and prebiotics on biological markers of these disorders, the findings indicate that advances in this field could be of value in the struggle against obesity and its associated-metabolic disorders.

## 1. INTRODUCTION

Obesity is a major public health concern affecting both the developed and the developing world. The obesity epidemic started to grow in US in the 1980s, with values rising from 22.9% obese adults in 1988–1994 to 30.5% in 1999–2000 [1]. In 1996, the World Health Organization (WHO) together with national Ministries of Health agreed to tackle obesity worldwide, but since then it has increased sharply, reaching values of at least 20% obese adults in most US states and European countries [2]. Obesity is detrimental to the quality of life and implies high health costs as a consequence of its associated morbidities. Overweight and obesity constitute risk factors for a number of chronic diseases including diabetes, cardiovascular diseases, nonalcoholic fatty liver disease, cancer, and other immune-related disorders such as asthma and infections [3].

Obesity results from a long-term positive imbalance between energy intake and expenditure with excessive increase in body fat. Obesity and the associated disorders are also characterized by a state of chronic, low-grade inflammation with abnormal cytokine and adipokine production [4]. Production of inflammatory immune mediators such as tumor-necrosis factor (TNF)-*α*, interleukin (IL)-6, IL-1*β*, CC-chemokine ligand 2 (CCL2 or monocyte chemotactic protein 1), and the proinflammatory adipokines leptin and resistin is usually high in these subjects, whereas production of the anti-inflammatory and insulin-sensitizing adipokine adiponectin is reduced [5]. Inflammation associated with obesity involves diverse signal transduction cascades including the nuclear factor kappa B (NF-*κ*B)/IKK*β* system and the Jun N-terminal kinase (JNK) [4, 6]. Leptin is the dominant long-term signal informing the brain of energy stores and, together with insulin, is secreted upon ingestion thus inhibiting food intake. However, human obesity is not commonly associated with leptin-deficiency but with leptin-resistance and increased levels of this adipokine. Leptin seems to exert a proinflammatory effect by inducing the production of CCL2, proinflammatory cytokines (TNF-*α*, IL-6, and IL-12), and also typical T helper (Th) 1-cytokines (IL-2 and IFN*γ*) involved in other chronic inflammatory and autoimmune disorders such as Crohn's disease [6, 7].

Although susceptibility to definitive increases in body weight is genetically determined, the environment also influences weight gain considerably. It is currently believed that macrosocial changes associated with regular intake of energy-dense foods and low-physical activity have created an obesogenic environment worldwide, constituting the cornerstone of the global obesity epidemic [8]. Traditional treatments based on calorie-restricted diets and increased physical activity have succeeded in controlling obesity to some extent [9]. Nevertheless, these strategies usually yield limited and short-lived weight reductions and most people regain some of their weight loss [3]. Neither has pharmacological therapy fully succeeded in effectively treating obesity for long-term periods and also has a number of side-effects [3, 10]. In this scenario, the identification of additional environmental factors involved in energy regulation is critical with a view to develop more efficient intervention strategies.

The human gut is populated by an array of bacterial species that coevolve with the host since birth and maintain dynamic interactions with each other throughout life. The collective genome (microbiome) of the gut microbiota contains at least 100 times as many genes as the human genome, with most serving human physiological functions [11]. The metabolic role of the gut microbiota is essential to the biochemical activity of the human body, resulting in salvage of energy, generation of absorbable compounds, and production of vitamins and other essential nutrients [12]. Thus, humans are considered superorganisms whose metabolism represents the combination of both microbial and human features [11]. The gut microbiota also regulates many aspects of innate and acquired immunity, protecting the host from pathogen invasion and chronic inflammation [13, 14]. In contrast, imbalances in the composition of gut microbiota have been associated with susceptibility to infections, immune-based disorders, and recently also with insulin resistance and body weight gain [15]. In the last decades, sound relationships between the composition of the gut microbiota and human health have been established, leading to the design of dietary strategies to favor the prevalence of beneficial bacteria to maintain a healthy status. These strategies include the administration of prebiotic oligosaccharides, which stimulate the growth and/or metabolic activity of beneficial bacteria, and also of selected bacterial strains (probiotics) in the form of functional foods and supplements [16]. Herein, the current knowledge of the relationships between the composition and functions of the gut microbiota and obesity is reviewed, including some studies intended to evaluate the effects of probiotics and prebiotics in the management of metabolic disorders.

## 2. GUT MICROBIOTA COMPOSITION, DIET, AND OBESITY

Obesity has been associated with increases in the relative abundance of *Firmicutes* and proportional reductions in *Bacteroidetes* by comparisons between the distal gut microbiota of genetically obese (leptin deficient *ob/ob* mice) and lean mice, as well as of that of obese and lean human subjects [17, 18]. In addition, obese human adults submitted to a hypocaloric diet (either low carbohydrate- or low fat-containing diet) showed significant increases in fecal proportions of *Bacteroidetes* paralleled to weight loss over a one-year-long intervention in a few subjects [18]; nonetheless, species diversity was reported to remain constant. Studies on the cecal microbiota of genetically obese mice and their lean littermates also related a higher proportion of *Archaea* to obesity [17]. These relationships between obesity and the gut microbiota composition were first based on DNA sequence analysis of the total distal gut microbiome of mice and humans obtained from genomic libraries or directly by pyrosequencing. Of these microbial groups, *Bacteroidetes* and *Firmicutes* constitute the dominant bacterial subdivision (>99%) among the 70 bacterial subdivisions identified in distal gut, while *Methanobrevibacterium smithii* constitutes the most prominent methanogenic archaeon among the 13 *Archaea* divisions reported to date based on 16S ribosomal DNA sequencing data [11]. More recently, diet-induced obesity in animal models has been associated with increases in the proportion of a single-uncultured clade within the *Mollicutes* class of *Firmicutes*, which was also diminished by subsequent dietary manipulations to limit weight gain, showing more specific relationships between obesity and components of the gut microbiota [19]. A study of a Chinese family, comprising 3 males and 4 females, also related the lowest *Bacteroidetes* to *Firmicutes* ratio to the overweight individual, and demonstrated a high degree of interpersonal variation in this value, ranging from 0.26 to 1.36 [20].

Differences in fecal microbiota composition were shown to predict overweight in children early in life. Children maintaining normal weight showed a greater number of bifidobacteria, while children becoming overweight showed a greater number of *Staphylococcus aureus* in feces during infancy [21]. Although the selected population group included children prone to allergy, who may show the described microbial aberrancies, the obtained results are also in accordance with the protective role attributed to breast-milk against developing obesity later in life [22], and the predominance of bifidobacteria in the gut of breastfed babies [23]. Shifts in composition of animal and human gut bacteria in response to dietary changes (a high protein/low carbohydrate or high-fat intake) have also been shown to alter microbial composition and activity in the large intestine that, in turn, could exert an impact on health [15, 24]. Obese humans submitted to a dietary intervention, based on reducing carbohydrate intake and increasing protein intake, showed reductions in populations of *Bifidobacterium*, and *Roseburia* spp. and *Eubacterium rectale* subgroups of clostridial cluster XIVa when carbohydrate intake was decreased, while no differences were detected in *Bacteroide*s or other clostridial clusters [24]. The abundance of *Roseburia* spp. and *E. rectale* group correlated well with the decline in fecal butyrate as carbohydrate intake was reduced; however, relationships to body weight were not established. Recent studies on the evolution of mammals and their gut microbes pointed out that the acquisition of a new diet is a fundamental driver for changes in gut bacterial diversity, which increases from carnivory to omnivory to herbivory [25]. Alterations in gut microbiota composition associated with genetic or diet-induced obesity have also been shown to be reversible by oral transfer of the gut microbiota from lean mice to a germ-free recipient [19, 26] or by administration of prebiotic substrates to animal models at least over short-term periods [27]. Therefore, it seems likely that a combination of environmental (e.g. diet) and genetic factors contributes to defining unique combinations of bacteria within an individual, which could favor either an obese or lean phenotype. In this context, some authors argue that both antibiotics and probiotics have demonstrated to act as growth promoters when used in animal feeding and, therefore, could contribute to current human obesity [28]. However, while antibiotics reduce gut microbiota populations, probiotics restores their levels. Therefore, their common effect on animal weight gain can be only a consequence of their common role in preventing infections. By contrast, other scientists consider that the intentional manipulation of the composition of gut microbiota via dietary strategies is a possible tool to revert or prevent overweight and particularly metabolic-associated disorders [19, 26, 27]. Although this line of research is still in its infancy, in the following sections we summarize current evidence on the mechanisms of action of gut microbiota on metabolic and immune aspects of obesity and the consequences of its dietary manipulation by pro- and prebiotics.

## 3. INFLUENCE OF THE GUT MICROBIOTA ON ENERGY METABOLISM

**Figure 1:**
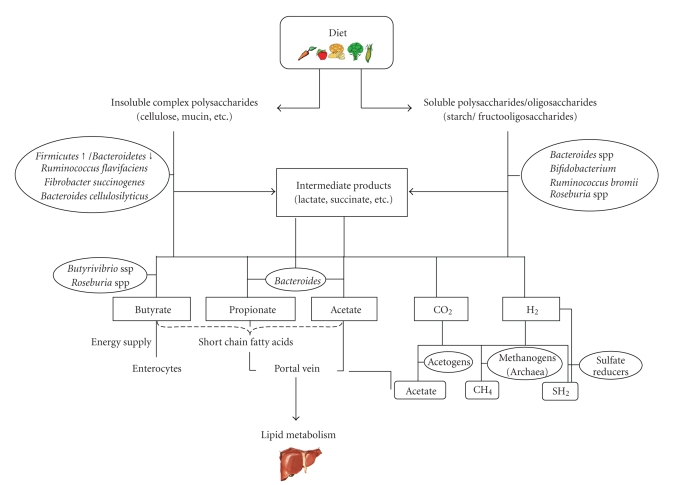
Schematic diagram of the main metabolic pathways of dietary poly- and oligosaccharides in the gut ecosystem.

The gut microbiota is considered a critical factor, together with lifestyle, involved in energy metabolism and obesity. Germ-free mice colonized by the distal gut microbiota of conventionally raised mice produced a remarkable increase (60%) in body fat within 10–14 days, although feed consumption was reduced compared to the control germ-free mice [29]. This process also stimulated the synthesis of leptin, and produced faster glycemia and insulinemia, paralleled to body-fat increase [29]. The microbial colonization was demonstrated to increase the host's ability to both harvest energy from the diet and store this energy in adypocites. This is thought to be achieved by diverse mechanisms including improvement of diet macronutrient utilization, generation of metabolites involved in energy balance and regulation of host gene expression. Commensal bacteria have specialized sets of hydrolyses and transporters to digest nutrients, like complex polysaccharides, that would, otherwise, be inaccessible to humans. These are the main energy sources for bacteria colonizing the large intestine and confer them a competitive advantage over transient bacteria. The microbial fermentation of undigested dietary compounds can provide approximately 10% of the daily energy supply in omnivores and up to 70% in herbivores [30]. The degradation of matrix and other dietary polysaccharides (xylans, manans, pectins, starch, and inulin) as well as host mucins lead to the generation of intermediate products (lactate, succinate, etc.) and finally short-chain fatty acids (SCFA), including butyrate, acetate, and propionate, which are almost completely absorbed along the gastrointestinal tract (Figure 1). The ability to degrade highly insoluble polymeric substrates, such as cellulose and mucin, seems to be limited to a subset of primary microbe degraders in the large intestine and requires the expression of specific substrate attachment, degradation, and uptake systems like the so-called cellulosome complex. In fact, cellulolytic species have been shown to form biofilm associations with plant surfaces in vitro, integrated by a higher fraction of *Firmicutes* and a smaller fraction of *Bacteroides,* which suggests a more prominent role of the former bacterial group in energy harvest from the diet by facilitating complex polysaccharide utilization [31]. Several clostridial clusters of *Firmicutes* are important butyrate-producing bacteria in the distal gut, such as *Roseburia, E. rectale, Eubacterium halli,* and *Anaerostipes caccae,* most of which are included in clostridial cluster XIVa [31]. Acetogenesis is another metabolic attribute of relevance to obesity identified in this clostridial cluster [31], which could partly explain the inverse relationship between *Firrmicutes* and body weight reductions in previous human intervention studies [18]. Soluble and less complex oligosaccharides such as starch and fructooligosaccharides can be utilized by other gut microbes such as *Bacteroides* and *Bifidobacterium,* which could also contribute to the generation of intermediary metabolites and finally to SCFA (Figure 1).

Although butyrate-producing bacteria would appear to be related to higher gut metabolic activity leading to overweight, butyrate is extensively utilized by enterocytes and generally regarded as a healthy metabolite [32]. The main role of butyrate is to fuel enterocytes, covering up to 70% of their energy needs and contributing to epithelial cell growth regulation and differentiation (Figure 1). Butyrate also exerts anti-inflammatory effects and seems to contribute to glucagon-like peptide 1 (GLP-1) generation, which is involved in satiety, by promoting differentiation of stem cells into positive GLP-1 L cells. Altogether, this may have beneficial effects on obese-prone subjects [32, 33]. Unlike butyrate, acetate and propionate generated in intestinal lumen can reach the blood stream and the liver through the portal vein (Figure 1). Acetate can contribute to lipid and cholesterol synthesis in the liver by activating the cytosolic acetyl S CoA synthetase 2, while propionate may inhibit lipid synthesis from acetate at least in rat hepatocytes [34]. In fact, high-propionate production through fermentation of starch or fructans has been associated with serum and liver cholesterol reduction in rats and the acetate to propionate ratio in portal blood, proposed as a possible maker of the effects of these dietary ingredients on lipid metabolism [35, 36]. Nevertheless, acetate administered at a high dose to rats and rat hepatocytes also induced AMP kinase and/or reduced SREBP-1c expression related to lipogenesis inhibition, therefore further studies should be carried out in humans to verify its positive or negative influences on lipid metabolism [37].

In addition to SCFA, hydrogen is produced by polysaccharide-degrading species and its further utilization by methanogens, acetogens and sulfate-reducing gut microbes also activates the metabolism and growth of polysaccharide-degrading bacteria (Figure 1). *Archaea*, which are the main gut methanogenic microorganisms, were also overrepresented in genetically obese mice as compared to their lean littermates and were related to a greater capacity to promote adiposity when transferred to germ-free recipients [38]. *Eubacterium dolichum*, a human *Mollicute*, was also shown to favor import and processing of simple sugars in subjects under a Western-style diet, partly explaining its association and that of *Firmicutes* division with obesity [19]. In addition, cross-feeding mechanisms between components of the gut microbiota have been identified at different stages of the utilization of complex energy-rich polysaccharides. Thus, *B. adolescentis* can degrade starch, generating intermediate products (lactate and acetate) that can be utilized by butyrate-forming bacteria such as *E. hallii* to generate butyrate [39] or by other intestinal bacterial groups that convert lactate into propionate by the acrylate pathway [40]. Coinoculation of *M. smithii* and *B. thetaiotaomicron* into germ-free mice showed that *M. smithii* directs *B. thetaiotaomicron* to focus on fermentation of dietary fructans to acetate, whereas *B. thetaiotaomicron*-derived formate is used by *M. smithii* for methanogenesis. Moreover, *B. thetaiotaomicron-M. smithii* cocolonization produced a significant increase in host adiposity compared with monoassociated, or *B. thetaiotaomicron-D. piger* biassociated animals [41]. These studies emphasize the role of interactive sets of microbes, rather than the role of individual microorganism within the gut ecosystem in energy-metabolism and body weight regulation. This makes it far more complex to identify those that are critical to obesity control through dietary strategies.

The gut microbiota may also influence energy balance by modifying gene expression of host-related metabolic functions. Angiogenesis, which is primarily involved in distributing nutrients to peripheral tissues, was shown to depend on the gut microbial colonization process. Although capillary network formation was arrested in adult germ-free mice, this developmental process restarted and was completed within 10 days after colonization with a complete microbiota harvested from conventionally raised mice, or with *Bacteroides thetaiotaomicron* [42]. Commensal bacteria, such as *B. thetaiotaomicron,* have also been shown to induce expression of host monosaccharide transporters in monocolonized mice [43]. This would lead to increasing the absorption of monosaccharides and SCFA and, thereby, promote the novo synthesis of lipids in the liver. In addition, the microbial colonization of germ-free mice increased liver expression of two key enzymes involved in the de novo fatty acid biosynthetic pathways, acetyl-CoA carboxylase and fatty acid synthase, as well as the transcriptional factors ChREBP and SREBP-1, which are involved in hepatocyte lipogenic responses to insulin and glucose [29]. Unlike colonized mice, germ-free animals were protected against the obesity that develops after consuming a Western-style, high-fat, sugar-rich diet by increasing fatty acid metabolism via two complementary mechanisms: (i) increasing levels of circulating fasting-induced adipose factor (Fiaf), which inhibits lipoprotein lipase thereby limiting fat storage in adipocytes and promoting fat oxidation in muscle; and (ii) increasing skeletal muscle and liver levels of phosphorylated AMP-activated protein kinase and its downstream targets, involved in fatty acid *β* oxidation [44].

Commensal gut microbiota and probiotics could also regulate serum lipids by taking part in bile acid metabolism. Bile salts are highly effective detergents that promote solubilization and absorption of dietary lipids throughout the intestine. The major bile salt modifications of microbial origin in the human gut include deconjugation, oxidation of diverse hydroxyl groups and 7 *α*/*β*-dehydroxylation [45]. Certain probiotics have been shown to decrease serum cholesterol levels by means of their bile salt hydrolytic activity [46]. Significant bile salt hydrolysis occurring in the proximal and terminal ileum reduces bile salt uptake through high-affinity transport system and lipid solubilization. This also leads to an increase in bile-acid excretion in feces and bile-acid synthesis from cholesterol [45]. For example, administration of *L. acidophilus* ATCC 43121 seemed to reduce serum cholesterol levels by bile acid deconjugation and dehydroxylation reactions during cholesterol metabolism in hypercholesterolemia-induced rats [46]. This intervention resulted in increased excretion of total fecal acid sterols and secondary bile acids (deoxycholic and lithocholic acids), and a reduction of primary bile acids (cholic and chenodeoxycholic acids). Particularly, the reduction in blood cholesterol levels was related to the increase in the insoluble bile acid, lithocholic acid. More recently, metabolomic studies have indicated that supplementation of *Lactobacillus paracasei* NCC2461 or *Lactobacillus rhamnosus* NCC4007 probiotics to germ-free mice colonized with human baby flora-induced changes in hepatic-lipid metabolism and enterohepatic recirculation of bile acids that led to a decrease in the plasma concentrations of lipoproteins VLDL and LDL, when compared to controls [47]. *Lactobacillus* supplementation also decreased fecal excretion of bile acids probably due to their accumulation in *Lactobacillus* probiotic cells. Probiotic administration also led to reductions of acetate in cecal content as well as of the hepatic acetate to propionate ratio, which was related to a reduction in serum lipids [47]. Furthermore, studies in vitro indicated that fecal commensal bacteria, but not probiotics, were able to reduce cholesterol to coprostanol and thus increasing its excretion in feces [48].

Some probiotic strains of the genus *Lactobacillus* and *Bifidobacterium* were also reported to synthesize conjugated linolenic acid (CLA) from polyunsaturated fatty acids of soy oil, which reduces serum lipids and cholesterol in liver. One example of these bacteria is *Lactobacillus rhamnosus* PL60, which is a human isolate that produces t10, c12-conjugated linoleic acid and was found to exert an antiobesity effect on diet-induced obese mice after 8 weeks of feeding. This strain reduced body weight without reducing energy intake, and caused a specific reduction of white adipose tissue without producing liver steatosis, which is a common side effect of CLA [49].

## 4. IMMUNE ROLE OF THE GUT MICROBIOTA AND OBESITY

**Figure 2:**
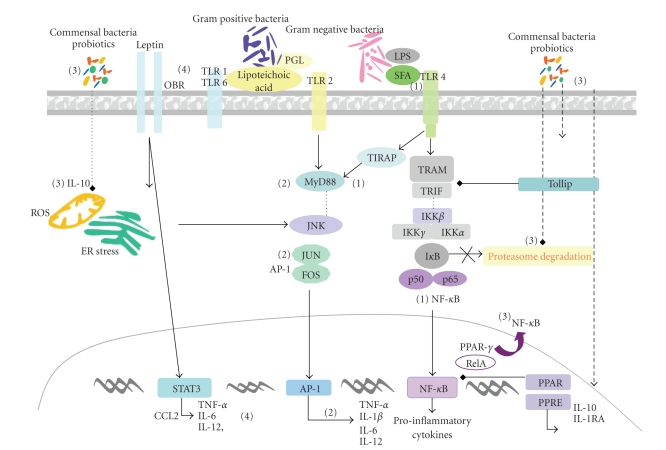
Schematic diagram of signaling pathways triggered by bacterial components, saturated fatty acids, and adipokines in epithelial and innate immune cells leading to either activation or negative regulation of proinflammatory pathways related to obesity and insulin resistance. (1) Lipopolysaccharide (LPS) from Gram-negative bacteria and saturated fatty acids (SFAs) is recognized by toll-like receptor (TLR) 4 activating proinflammatory pathways involving the MyD88 (myeloid differentiation primary-response protein 88)-dependent and -independent pathways that may lead to activation of nuclear factor (NF)-*κ*B and activator protein-1 (AP-1) with production of pro-inflammatory cytokines. (2) Peptidoglycan (PGL) and lipoteichoic acid from Gram-positive bacteria are recognized by TLR-2 triggering the activation of the MyD88-dependent pathway. (3) Commensal bacteria and some probiotics may suppress activation of NF-*κ*B cascade by (i) promotion of nuclear export of NF-*κ*B subunit relA in complex with PPAR-*γ*; (ii) inhibition of I*κ*B ubiquitination and degradation, (iii) induction of anti-inflammatory (IL10) cytokine production. (4) Leptin interacts with its receptors (OBR) activating the signal transducer and activator of transcription (STAT), and induces production of CCL2, proinflammatory cytokines, and reactive oxygen species (ROS) causing endoplasmic reticulum (ER) stress.

Obesity is considered an inflammatory disorder, which affects both innate and adaptive immunity and favors the development of other disorders such as type-2 diabetes and cardiovascular diseases [50]. In fact, chronic activation of innate immunity is regarded as a risk factor as it favors the development of these disorders, which could also be influenced by the gut microbiota [27, 51]. The gut microbiota largely regulates innate and adaptive immunity, influencing local and systemic responses (Figure 2). The recognition of bacterial components through pattern-recognition receptors (PRRs), such as toll-like receptors (TLRs) of innate immune cells, is considered to be the starting point of immunity, informing the immunocompetent cells to respond properly to each environmental stimulus (e.g., pathogens or harmless microbes) [13]. TLR-4 recognizes lipopolysaccharide (LPS) from Gram-negative bacteria, while TLR-2 recognizes lipopeptides and lipoproteins from various pathogens, and peptidoglycan and lipoteichoic acid from Gram-positive bacteria (Figure 2) [52]. Upon ligand binding, TLR interacts with different adaptor proteins (MyD88, TIRAP/Mal, TRIF, and TRAM) activating the transcription of different downstream effector systems, such as the mitogen-activated kinases (MAPK), the NF-*κ*B/IKK*β* system, and the activator protein-1 (AP-1) with production of cytokines and diverse immune mediators [53]. Cytokines such as TNF-*α*, IL-1*β*, and IL-6 are the major proinflammatory mediators produced in response to TLR-4 stimulation by endotoxin (LPS) as well as those increased in obese and insulin-resistant patients (Figure 2) [54]. Unlike pathogenic microbes, commensal bacteria maintain a peaceful relationship with their hosts by producing a transient activation of the NF-*κ*B cascade or its suppression by diverse mechanisms including (i) promotion of nuclear export of NF-*κ*B subunit relA in complex with PPAR-*γ* [55], (ii) inhibition of I*κ*B ubiquitination and degradation in epithelial cells [56], (iii) regulation of TLR expression and upregulation of the negative regulator Tollip protein [57], and (iv) induction of anti-inflammatory cytokines such as IL-10 [58]. TLRs and derived cytokines also play a pivotal role in linking innate and adaptive immunity through exerting action on T-cells and particularly on dedritic cells (DCs), keeping a physiological Th1/Th2 balance [13]. Th1-polarized responses characterized by overproduction of IFN-*γ*, IL-2, and IL-12 cytokines are associated with clearance of intracellular pathogens as well as with chronic diseases including diabetes and obesity. Most TLR-activated DCs induce differentiation of naïve CD4+ T cells into Th1 cells, while TLR2-activated DCs promote the differentiation of Th2-cells or regulatory T cells by producing high levels of anti-inflammatory cytokine IL-10 which could help to counteract the inflammatory status associated with obesity [58]. Interestingly, TLRs have been identified not only in innate and adaptive immune cells but also in insulin-responsive tissues such as the adipose tissue, muscle, and liver, suggesting a connection between immunity, microbial stimulation, and metabolism [59]. Diet-induced and genetically obese mice (*ob/ob* or *db/db*) showed a significant upregulation of expression of TLR-1 to -9 in adipocytes and preadipocytes along with higher cytokine production upon stimulation [60]. In particular, it is known that TLR-4 can be activated by both lipopolysaccharide (LPS) and dietary-saturated fatty acids inducing upregulation of common intracellular inflammatory pathways, such as JNK and NF-*κ*B in adipocytes and macrophages, related to the induction of insulin resistance and increased adiposity (Figure 2) [51]. Conversely, adipocyte-specific knockdown of TLR4 prevented cytokine expression induced either by LPS or saturated fatty acids and similar effects were shown in macrophages. With some exceptions, loss-of-functional mutation in TLR-4 also prevented diet-induced obesity and insulin resistance in vivo mice models [51]. Recently, metabolic endotoxaemia, characterized by an increase in serum LPS levels, has been demonstrated to be an inflammatory factor, causative of body weight gain, insulin resistance, and diabetes in high-fat fed animal models [27, 61]. In contrast, the inhibition of the gut microbiota by antibiotic administration (norfloxacin and ampicillin) in two different mouse models of insulin resistance resulted in reduced serum LPS levels, low-grade inflammation, obesity, and type-2 diabetes, demonstrating the link between the gut microbiota and certain metabolic disorders [15]. LPS stimulation also produces a cytokine-mediated increase in plasma lipid levels by increasing the synthesis of VLDL lipoproteins in the liver and inhibiting lipoprotein lipase. In fact, mobilization of lipid stores is considered a mechanism to fuel the host's response against infections; moreover, lipoproteins also seem to help fight against infection by binding and neutralizing LPS [62]. Therefore, common responses can be induced by “pathogenic lipid nutrients” and microorganisms mainly related to TLR-4-signaling and proinflammatory cytokine and gene transcription activation pathways. In this scenario, one can hypothesize that shifts in gut microbiota composition caused by a high-saturated fatty acid-containing diet [27], together with dietary lipids, could constitute synergic TLR signals, thus contributing to the amplification of inflammation occurring in obesity. Consequently, it has been suggested that probiotics and prebiotics with anti-inflammatory properties could be of help in the fight against obesity and associated disorders, as reported in other chronic inflammatory diseases [53]. Although few specific studies have proven such a hypothesis so far, the administration of the probiotic VSL3# was demonstrated to exert a preventive effect against type-1 diabetes in a nonobese diabetic mice model by immunomodulatory mechanisms, inducing IL-10 production in Peyer patches, and spleen and its expression in the pancreas [63]. In addition, *Lactobacillus* culture-supernatants were shown to reduce in vitro leptin production by adipocytes, thereby reducing IFN-*γ* production by lymphocytes and exerting an anti-inflammatory role [64]. Oral administration of a functional food product containing *L. plantarum* 299v to heavy smokers for six weeks led to a decrease in leptin, systolic blood pressure, and fibrinogen, which was attributed to the anti-inflammatory effects of this probiotic, suggesting it would be able to reduce cardiovascular risk [65]. In contrast, oral administration of *Lactobacillus acidophilus* and *Bifidobacterium longum* strains to human subjects did not influence serum leptin levels [66]. The administration of a prebiotic (oligofructose) to high-fat-diet fed mice was also shown to restore *Bifidobacterium* levels, which positively correlated with improved glucose tolerance, glucose-induced insulin secretion, and normalization of inflammatory tone by decreasing endotoxaemia in plasma and proinflammatory cytokines in adipose tissue [27].

## 5. CONCLUSIONS AND FUTURE CHALLENGES

Gut microbes are viewed as novel factors involved in host physiology and body weight regulation by driving a number of metabolic and immune functions. The initial association of the microbial colonization process of the germ-free intestine with body weight gain conferred a negative role to gut microbes with respect to obesity. Further, relationships established between a specific microbiota structure and a lean or obese phenotype have suggested that different microbes may influence body weight differently, and species- and stain-specific functions are being defined. In addition, endotoxaemia and dysbiosis have been identified as inflammatory factors responsible for insulin resistance and body weight, thereby returning to the concept that a healthy microbiota may be beneficial in preventing these disorders. Although the cause-effect relationships of the gut microbiota with obesity remain unclear and a limited number of in vivo trials have been done to assess the effects of specific microbial strains (commensals and probiotics), and prebiotics on metabolic disorders, the knowledge provided by these studies constitutes a breakthrough in the identification of their etiology. Further work based on systems biology coupled with “omic” technologies (metagenomics, trancriptomics, and metabolomics) will be critical to shed light on the roles of specific sets of microbes on metabolic disorders, with a view to design more efficient dietary-based strategies to reduce their risk.
